# Effects of Zibotentan Alone and in Combination with Dapagliflozin on Fluid Retention in Patients with CKD

**DOI:** 10.1681/ASN.0000000000000436

**Published:** 2024-07-12

**Authors:** J. David Smeijer, Victor S. Wasehuus, Neeraj Dhaun, José Luis Górriz, Maria José Soler, Magnus Åstrand, Anne-Kristina Mercier, Peter J. Greasley, Phil Ambery, Hiddo J.L. Heerspink

**Affiliations:** 1 Department of Clinical Pharmacy and Pharmacology, University Medical Center Groningen, University of Groningen, Groningen, The Netherlands; 2 Steno Diabetes Center Copenhagen, Herlev, Denmark; 3 BHF/University Centre for Cardiovascular Science, University of Edinburgh, Edinburgh, United Kingdom; 4 Department of Nephrology, University Clinical Hospital, INCLIVA Research Institute, University of Valencia, Valencia, Spain; 5 Nephrology Department, Vall d’Hebron University Hospital, Vall d’Hebron Institute of Research, Barcelona, Spain; 6 Clinical Pharmacology and Quantitative Pharmacology, Clinical Pharmacology and Safety Sciences, R&D, AstraZeneca, Gothenburg, Sweden; 7 George Institute for Global Health, Barangaroo, New South Wales, Australia

**Keywords:** CKD, heart failure, pharmacokinetics, SGLT2, pharmacology

## Abstract

**Key Points:**

- Increasing doses of the endothelin receptor antagonist zibotentan and lower eGFR were associated with a higher risk of fluid retention.
- The higher risk of fluid retention could be attenuated by the combination of zibotentan with the sodium-glucose cotransporter 2 inhibitor dapagliflozin.

**Background:**

Endothelin receptor antagonists (ERAs) reduce albuminuria but are limited by fluid retention risk, particularly in patients with CKD. Combining ERAs with sodium-glucose cotransporter 2 (SGLT2) inhibitors, which have diuretic effects, offers a promising strategy to mitigate fluid retention. In this *post hoc* analysis of the Zibotentan and Dapagliflozin for the Treatment of CKD (ZENITH-CKD) trial, we assessed fluid dynamics in patients with CKD treated with the ERA zibotentan alone and in combination with the SGLT2 inhibitor dapagliflozin.

**Methods:**

In the ZENITH-CKD trial, 508 patients with CKD (eGFR ≥20 ml/min per 1.73 m^2^ and a urinary albumin-creatinine ratio of 150–5000 mg/g) were randomized to treatment with placebo, dapagliflozin 10 mg plus placebo, zibotentan (0.25, 1.5, or 5 mg) plus dapagliflozin 10 mg, and zibotentan 5 mg plus placebo. We evaluated correlations between changes in fluid retention markers and bioimpedance-measured extracellular fluid in response to zibotentan treatment. We used Cox proportional hazards regression to assess the association between zibotentan/dapagliflozin treatment, baseline characteristics, and fluid retention and the relationship between zibotentan plasma exposure and fluid retention.

**Results:**

After 3 weeks of treatment with zibotentan 0.25, 1.5, or 5 mg plus dapagliflozin 10 mg, changes in body weight (*β*=0.36 [95% confidence interval (CI), 0.26 to 0.45]) per kg, B-type natriuretic peptide (*β*=0.38 [95% CI, 0.22 to 0.54]) per doubling, and hemoglobin (*β*=−0.29 [95% CI, −0.48 to −0.10]) per g/dl were independently associated with changes in extracellular fluid. Higher doses of zibotentan were associated with significantly higher risk of fluid retention compared with dapagliflozin alone (zibotentan 5 mg hazard ratio (HR) 8.50 [95% CI, 3.40 to 21.30]). The HR attenuated when zibotentan was combined with dapagliflozin (zibotentan/dapagliflozin 5/10 mg HR 3.09 [95% CI, 1.08 to 8.80], zibotentan/dapagliflozin 1.5/10 mg 2.70 [95% CI, 1.44 to 5.07], and zibotentan/dapagliflozin 0.25/10 mg HR 1.21 [95% CI, 0.50 to 2.91]). The risk of fluid retention was higher with higher zibotentan exposure and lower eGFR.

**Conclusions:**

High doses of zibotentan were associated with a higher risk of fluid retention, which was attenuated with lower doses and the addition of dapagliflozin.

**Clinical Trial registry name and registration number:**

ZENITH-CKD Trial, NCT04724837.

## Visual Abstract

**Figure:**
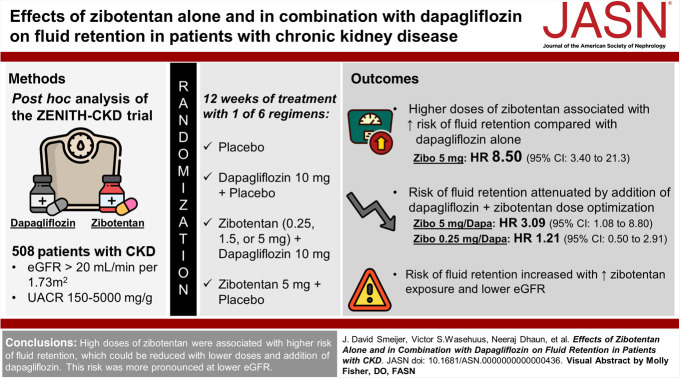

## Introduction

Over the past few years, new pharmacotherapeutic options to slow progression of CKD have emerged, including sodium-glucose cotransporter 2 (SGLT2) inhibitors and the mineralocorticoid receptor antagonist finerenone.^1^ Despite these new evidence-based therapies, a significant proportion of patients with CKD continue to have elevated albuminuria, which is associated with a persistently higher risk of progressive kidney function loss and cardiovascular complications.^2^

Endothelin receptor antagonists (ERAs) represent another pharmacological treatment proven to reduce the rate of kidney function decline in type 2 diabetes and IgA nephropathy.^3,4^ The phase 2B Zibotentan and Dapagliflozin for the Treatment of CKD (ZENITH-CKD) dose-finding trial showed that the addition of the highly selective ERA zibotentan in combination with the SGLT2 inhibitor dapagliflozin also significantly reduced albuminuria compared with dapagliflozin alone, suggesting complementary mechanisms.^5^

Although ERAs are potent therapies to reduce albuminuria and slow CKD progression, their clinical development has been hampered because of the risk of heart failure due to fluid retention, in particular in individuals with CKD who may benefit most from these therapies. Because SGLT2 inhibitors have osmotic diuretic properties, combining a SGLT2 inhibitor with a selective ERA holds promise to augment nephroprotection while potentially mitigating fluid retention. Indeed, in the ZENITH-CKD trial, adding low-dose zibotentan to dapagliflozin did not lead to additional fluid retention as measured by bioimpedance spectroscopy compared with dapagliflozin alone, while it significantly decreased albuminuria.^5^

Understanding how ERAs individually and in combination with SGLT2 inhibitors influence fluid dynamics in patients with CKD can inform the optimal use of zibotentan in clinical practice and guide the use of this therapy in patients who benefit most with minimal risk of side effects. We therefore performed an in-depth analysis of the ZENITH-CKD trial, first, to assess whether changes in surrogate measures of fluid retention, including B-type natriuretic peptide (BNP) and hematocrit, correlated with changes in bioimpedance-measured extracellular fluid (ECF) status during treatment to monitor fluid retention in clinical practice using clinically readily available markers; second, to identify clinical characteristics at baseline that predict the development of fluid retention in patients receiving zibotentan; and third, to evaluate whether zibotentan plasma exposure is associated with fluid retention to develop optimal dosing strategies for patients with CKD taking into account pharmacokinetic (PK) properties and kidney function.

## Methods

### Study Design

ZENITH-CKD is a randomized, double-blind, active-controlled, phase 2b, clinical trial. The study design and primary results have been published previously.^5,6^ The ZENITH-CKD trial was conducted in accordance with the Declaration of Helsinki, Guideline for Good Clinical Practice, and applicable local regulatory requirements. All participants provided written informed consent before study-specific procedures commenced. The trial is registered at ClinicalTrials.gov (NCT04724837). After randomization, participants attended study visits at weeks 1, 3, 6, 9, 12, and 14. The study medication was discontinued at week 12, and a follow-up visit was scheduled 2 weeks after discontinuation of study medication to assess off-drug effects.

### Participants

This study included adults aged 18–90 years diagnosed with CKD, defined by an eGFR ≥ 20 ml/min per 1.73 m^2^ and a urinary albumin-creatinine ratio (UACR) of 150–5000 mg/g. The use of an SGLT2 inhibitor or fixed-dose SGLT2 inhibitor combinations within 4 weeks before enrollment as comedication was not allowed. All participants were receiving a stable dose of an angiotensin-converting enzyme inhibitor or an angiotensin receptor blocker for at least 4 weeks before screening. In addition, all participants who were taking a diuretic before the start of the study were asked to keep the dose stable during the double-blind phase. Only a few participants initiated a diuretic during the study.^6^ In addition, patients with autosomal dominant or recessive polycystic kidney disease, an acute coronary syndrome event within 3 months before screening, type 1 diabetes, unstable heart failure requiring hospitalization, or elevated natriuretic peptides (BNP ≥200 pg/ml or *N*-terminal pro BNP [NT-proBNP] ≥600 pg/ml; BNP ≥400 pg/ml or NT-proBNP ≥1200 pg/ml if associated with atrial fibrillation) were excluded. A complete list of trial inclusion and exclusion criteria was previously published.^5^

### Procedures

Originally, the ZENITH-CKD trial used a two-part design. In part A, participants were randomized to placebo, dapagliflozin 10 mg/d plus placebo, zibotentan 5 mg plus dapagliflozin 10 mg/d, or zibotentan 5 mg/d. Part A was designed to assess the effect of zibotentan and dapagliflozin as single agents and their combination versus placebo. Part B introduced two additional arms: zibotentan 0.25 mg plus dapagliflozin 10 mg/d and zibotentan 1.5 mg plus dapagliflozin 10 mg/d. Part B was designed to assess the effect of various zibotentan doses in combination with a fixed dapagliflozin dose versus dapagliflozin alone.

Following advice from the data monitoring committee, randomization to the zibotentan 5 mg plus dapagliflozin and zibotentan alone treatment arms was stopped because the rate of fluid retention events made it unlikely that the target population of the trial would receive a positive benefit–risk balance. Developments in treatment guidelines has established SGLT2 inhibitors as standard-of-care treatment in CKD, and as a consequence, enrollment in the placebo arm was closed as well. Treatment allocation was updated to 2:1:2 for the remaining three arms: zibotentan 1.5 mg plus dapagliflozin, zibotentan 0.25 mg plus dapagliflozin, and dapagliflozin 10 mg.

At each visit, vital signs and body weight were measured, and blood and urine samples were collected for laboratory analysis. At each visit, markers of fluid retention, such as body weight, BNP, hemoglobin, and hematocrit, were measured in a central laboratory. A fluid retention end point was prespecified and defined as a change in body weight of at least 3% (at least 2.5% must have been from total body water) from baseline or an increase from baseline of at least 100% in BNP (and either a BNP concentration >200 pg/ml if without atrial fibrillation or BNP >400 pg/ml if with atrial fibrillation). Participants who developed fluid retention were discontinued from the study medication but continued to participate in study visits according to the protocol. We also assessed ECF volumes using bioimpedance spectroscopy (SOZO Body Composition Analyser [ImpediMed, Brisbane, QLD, Australia]) at all study visits.

### Pharmacokinetics

Predose (trough) plasma samples for PK analysis were collected at weeks 3 and 9. The predose samples were used to obtain the individual geometric mean predose zibotentan plasma concentration, which subsequently was used for graphical assessment of the plasma concentration in relation to eGFR and for exposure-fluid retention risk analysis.

### Statistical Analysis

The results presented here are based on data from all six ZENITH-CKD treatment arms—the three arms that formed the basis for the primary outcome analysis (dapagliflozin 10 mg, zibotentan 0.25 mg plus dapagliflozin, and zibotentan 1.5 mg plus dapagliflozin) and the three discontinued treatment arms (placebo, zibotentan 5 mg plus dapagliflozin, and zibotentan 5 mg). All randomized participants who underwent a study intervention were included in the analysis. The treatment arm allocation is based on the actual treatment participants received.

Summary statistics were used to describe the baseline clinical and demographic characteristics per treatment arm. To assess whether changes in surrogate measures of fluid retention, including body weight, BNP, NT-proBNP, hemoglobin, and hematocrit, correlated with changes in bioimpedance-measured ECF status among participants on zibotentan, first the change from baseline to week 3 was calculated for all markers. The week-3 time point was chosen to maximize detection of a direct effect of zibotentan on fluid retention and minimize the longer-term effect of dapagliflozin on body weight and hematocrit. We also compared change from baseline at week 3 in UACR and systolic BP with ECF status using Pearson correlation. Log transformations were applied to non-normally distributed variables: BNP, NT-proBNP, and UACR. We did not impute missing data (Supplemental Table 1). Pearson correlation coefficients were then computed between changes of fluid retention markers, using all pairwise complete observations. Subsequently, unadjusted linear regression was used to assess the association between the change in body weight and laboratory-based proxies of fluid retention and the change in bioimpedance-estimated ECF. Variables significantly associated with ECF changes were included in a multivariable linear regression model as well. To prevent collinearity between hematocrit and hemoglobin and between BNP and NT-proBNP, we only included the variable with the strongest correlation in unadjusted linear regression to the multivariable regression model.

**Figure 1:**
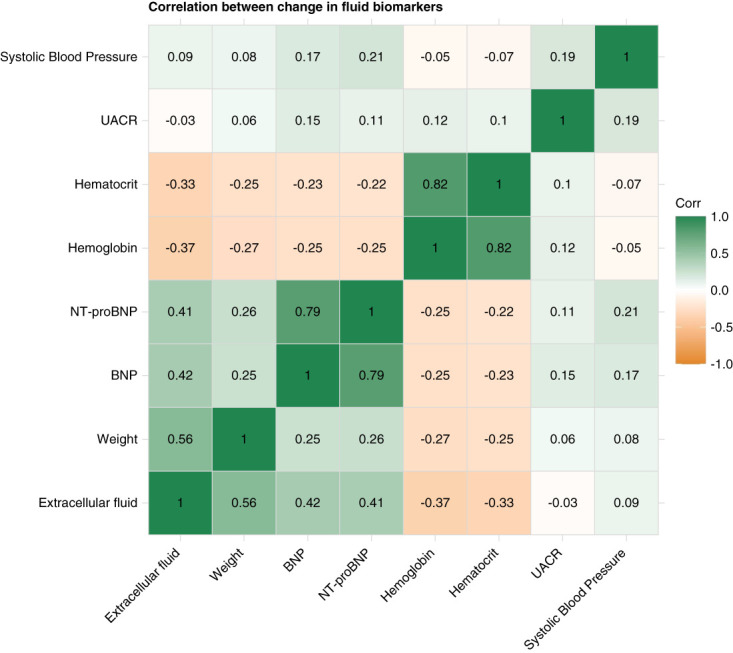
**Pearson correlation coefficient for change in selected biomarkers from baseline to week 3 only for ZENITH-CKD treatment arms involving zibotentan (0.25, 1.5, or 5 mg/d).** The change in biomarkers is expressed in their original units, except for BNP, NT-proBNP, and UACR where the log change from baseline was used. Original units: ECF=L; weight=kg; hemoglobin=g/dl; hematocrit=ratio; systolic BP=mm Hg. BNP, B-type natriuretic peptide; ECF, extracellular fluid; NT-proBNP, *N*-terminal pro B-type natriuretic peptide; UACR, urinary albumin-creatinine ratio; ZENITH-CKD, Zibotentan and Dapagliflozin for the Treatment of CKD.

Cox proportional hazards regression was used to assess the association between treatment and the relative hazard of the prespecified fluid retention event. The first model included age, treatment, body weight, systolic BP, eGFR, BNP, hemoglobin, type 2 diabetes status, and endothelin-1 (ET-1) as covariates. Second, because eGFR and hemoglobin were strongly correlated, we excluded hemoglobin as a covariate. For variables unavailable at baseline, we imputed missing values with measurements collected at the screening visit 4 weeks before baseline assessments were performed. Cox proportional hazards regression was also used to graphically display the risk of prespecified fluid retention event versus zibotentan predose plasma concentration and eGFR. The graphical display of risk versus zibotentan exposure was based on a Cox proportional hazards model. The model included zibotentan predose plasma concentration, a dichotomous variable for eGFR above or below 45 ml/min per 1.73 m^2^, and an interaction term between both variables as covariates. We also used a Cox proportional hazards model to determine the association between the risk of fluid retention and baseline eGFR for the different treatment arms. The model included baseline eGFR, treatment assignment, and an interaction term between both variables as covariates. All analyses were performed with the software package “R,” version 4.3.1. (R Foundation for Statistical Computing, Vienna, Austria).

## Results

Between April 28, 2021, and January 17, 2023, a total of 1492 participants were screened, of whom 508 participants were randomly assigned and received any study intervention. There were 21 participants (4%) allocated to placebo, 177 (35%) to the dapagliflozin group, 91 (18%) to the zibotentan 0.25 mg plus dapagliflozin group, 179 (35%) to the zibotentan 1.5 mg plus dapagliflozin group, 21 (4%) to the zibotentan 5 mg plus dapagliflozin group, and 19 (4%) to the zibotentan 5 mg group. During 12 weeks of treatment, 85 participants (17%) discontinued the study medication (Supplemental Figure 1).

### Baseline Characteristics

Baseline characteristics were balanced among treatment groups (Table 1). The mean age was 63 years (SD 12), 157 (31%) participants were female, 345 participants (68%) were White, and 302 participants (59%) were diagnosed with type 2 diabetes. The median UACR was 569 mg/g (interquartile range, 269–1182), and mean eGFR was 46 ml/min per 1.73 m^2^ (SD 21).

**Table 1:** Baseline demographics and clinical characteristics of participants in the Zibotentan and Dapagliflozin for the Treatment of CKD trial by treatment arm

Characteristic	Dapagliflozin 10 mg, *N*=177	Placebo, *N*=21	Zibotentan/Dapagliflozin 0.25/10 mg, *N*=91	Zibotentan/Dapagliflozin 1.5/10 mg, *N*=179	Zibotentan/Dapagliflozin 5/10 mg, *N*=21	Zibotentan 5 mg, *N*=19
Age, yr	64 (12)	63 (10)	61 (13)	63 (12)	62 (10)	65 (6)
Female sex, *n* (%)	55 (31)	8 (38)	28 (31)	55 (31)	7 (33)	4 (21)
**Race, *n* (%)**						
Asian	26 (15)	4 (19)	18 (20)	26 (15)	3 (14)	2 (11)
Black	22 (12)	3 (14)	7 (8)	17 (9)	1 (5)	4 (21)
Other	4 (2)	1 (5)	10 (11)	12 (7)	0 (0)	3 (16)
White	125 (71)	13 (62)	56 (62)	124 (69)	17 (81)	10 (53)
BMI, kg/m^2^	30 (5)	31 (6)	30 (5)	30 (5)	30 (5)	32 (4)
Systolic BP, mm Hg	138 (18)	143 (14)	136 (18)	136 (16)	138 (14)	131 (13)
Diastolic BP, mm Hg	80 (10)	83 (9)	80 (11)	79 (9)	82 (10)	78 (7)
Type 2 diabetes, *n* (%)	105 (59)	14 (67)	52 (57)	104 (58)	13 (62)	14 (74)
**CKD cause, *n* (%)**						
Chronic GN	20 (11)	4 (19)	10 (11)	25 (14)	1 (5)	2 (11)
Ischemic or hypertensive nephropathy	32 (18)	4 (19)	20 (22)	30 (17)	4 (19)	1 (5)
Other	18 (10)	1 (5)	6 (7)	17 (9)	1 (5)	0 (0)
Type 2 diabetes and CKD	93 (53)	12 (57)	44 (48)	88 (49)	14 (67)	14 (74)
Unknown	14 (8)	0 (0)	11 (12)	19 (11)	1 (5)	2 (11)
eGFR, ml/min per 1.73 m^2^	45 (21)	44 (11)	48 (23)	47 (23)	42 (11)	43 (10)
Median UACR, mg/g	577 (280–1151)	536 (423–1113)	527 (216–1275)	567 (239–1191)	635 (440–1107)	576 (421–906)
Cardiovascular disease history, *n* (%)	38 (21)	4 (19)	12 (13)	38 (21)	3 (14)	1 (5)
BNP, ng/L	36 (19–69)	50 (29–151)	37 (19–80)	35 (20–62)	47 (33–90)	41 (28–62)
NT-proBNP, pg/ml	15 (6–28)	24 (9–66)	16 (8–27)	14 (7–31)	22 (16–26)	17 (14–38)
Hemoglobin, g/dl	13.2 (1.7)	13.0 (1.9)	13.2 (1.6)	13.0 (1.6)	12.9 (1.5)	12.8 (1.5)
Hematocrit, ratio	0.40 (0.05)	0.39 (0.05)	0.40 (0.05)	0.39 (0.05)	0.39 (0.04)	0.39 (0.05)
Copeptin, ng/L	151 (99)	148 (64)	169 (123)	163 (111)	139 (72)	180 (123)
Sodium (plasma), mmol/L	140 (3)	139 (4)	140 (3)	140 (3)	139 (3)	139 (3)
Sodium (urine), mmol/L	90 (40)	84 (41)	89 (36)	94 (37)	94 (31)	94 (26)
Fractional excretion of sodium, %	1.5 (1.0–2.3)	1.4 (1.0–1.6)	1.6 (1.0–2.1)	1.7 (0.9–2.4)	1.7 (1.3–2.5)	1.7 (1.3–2.3)
ECF, %	48.4 (4.0)	48.5 (3.2)	48.4 (4.3)	48.1 (3.2)	48.2 (2.7)	48.0 (3.6)
Intracellular fluid, %	51.6 (4.0)	51.5 (3.2)	51.6 (4.3)	51.9 (3.2)	51.8 (2.7)	52.0 (3.6)
Total body water, L	42.8 (9.2)	44.9 (9.5)	41.6 (8.8)	44.1 (10.0)	43.3 (8.9)	45.0 (9.2)

For all continuous variables, the values between parentheses denote SD or interquartile range (urinary albumin-creatinine ratio, B-type natriuretic peptide, *N*-terminal pro B-type natriuretic peptide, fractional excretion of sodium). BMI, body mass index; BNP, B-type natriuretic peptide; ECF, extracellular fluid; NT-proBNP, *N*-terminal pro B-type natriuretic peptide; UACR, urinary albumin-creatinine ratio.

### Change in Fluid Retention Biomarkers

Changes in natriuretic peptides, body weight, and bioimpedance-measured ECF were all positively correlated with each other after 3 weeks of treatment with zibotentan. ECF change at 3 weeks was negatively correlated with changes in hematocrit and hemoglobin (Figure 1 and Supplemental Figure 2). In unadjusted linear regression analyses, changes in body weight, natriuretic peptides, hemoglobin, hematocrit, and ET-1 were all significantly associated with changes in ECF after 3 weeks of treatment with zibotentan (Table 2). Change in systolic BP or UACR after 3 weeks did not correlate with ECF or any other surrogate measure of fluid retention (Figure 1 and Supplemental Table 4). In multivariable linear regression analyses, changes in body weight, BNP, and hemoglobin remained statistically significantly associated with changes in ECF (total R^2^ 0.42; Table 2).

**Table 2:** Change in extracellular fluid (in liters) per change in selected fluid biomarkers after 3 weeks of zibotentan treatment in unadjusted and multivariable-adjusted linear regression models

Variable	Unadjusted	Multivariable
	Estimate (95% CI)	Estimate (95% CI)
Body weight change, per kg	0.47 (0.39 to 0.56)	0.36 (0.26 to 0.45)
BNP change, per doubling	0.61 (0.45 to 0.78)	0.38 (0.22 to 0.54)
NT-proBNP change, per doubling	0.58 (0.41 to 0.74)	NA
Hemoglobin change, per g/dl	−0.64 (−0.85 to −0.43)	−0.29 (−0.48 to −0.10)
Hematocrit change, per 0.1 L/L	−1.77 (−2.44 to −1.09)	NA
ET-1 change, per doubling	0.30 (0.01 to 0.61)	0.17 (−0.07 to 0.41)
Total adjusted R^2^		0.42

Variables included in the multivariable adjusted model: body weight, B-type natriuretic peptide, hemoglobin, and endothelin-1. BNP, B-type natriuretic peptide; CI, confidence interval; ET-1, endothelin-1; NA, not applicable; NT-proBNP, *N*-terminal pro B-type natriuretic peptide.

### Fluid Retention Events

**Figure 2:**
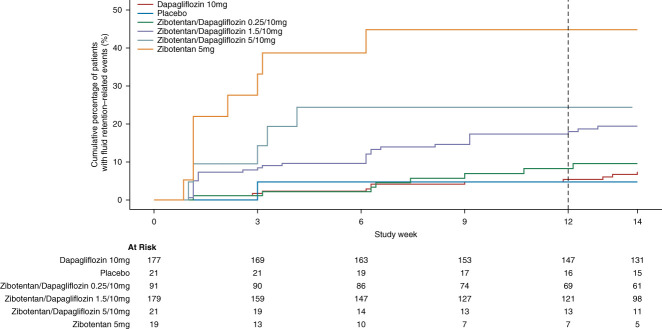
Kaplan–Meier curve of fluid retention events for all treatment arms.

In total, 69 participants (14%) experienced a prespecified fluid retention end point during the 12-week treatment period: 5% (1/21) in the placebo group, 8% (14/177) in the dapagliflozin plus placebo group, 9% (8/91) in the zibotentan 0.25 mg plus dapagliflozin group, 18% (33/179) in the zibotentan 1.5 mg plus dapagliflozin group, 24% (5/21) in the zibotentan 5 mg plus dapagliflozin group, and 42% (8/19) in the zibotentan 5 mg group. Participants who experienced the fluid retention end point were more likely to have a diagnosis of type 2 diabetes; had a significantly lower eGFR, hemoglobin, and hematocrit; and had a significantly higher BNP and NT-proBNP at baseline (Figure 2 and Table 3).

**Table 3:** Zibotentan and Dapagliflozin for the Treatment of CKD fluid biomarkers at baseline by fluid event status

Characteristic	Event=No, *N*=439	Event=Yes, *N*=69
Age, yr	63 (12)	65 (10)
Female sex, *n* (%)	137 (31)	20 (29)
**Race**		
Asian	70 (16)	9 (13)
Black	41 (9)	13 (19)
Other	24 (5)	6 (9)
White	304 (69)	41 (59)
**Treatment arm, *n* (%)**		
Dapagliflozin 10 mg	163 (37)	14 (20)
Placebo	20 (5)	1 (1)
Zibotentan/Dapagliflozin 0.25/10 mg	83 (19)	8 (12)
Zibotentan/Dapagliflozin 1.5/10 mg	146 (33)	33 (48)
Zibotentan/Dapagliflozin 5/10 mg	16 (4)	5 (7)
Zibotentan 5 mg	11 (3)	8 (12)
Weight, kg	85 (17)	87 (18)
eGFR, ml/min per 1.73 m^2^	47 (22)	41 (18)
BNP, ng/L	35 (19–62)	63 (30–95)
NT-proBNP, pg/ml	14 (7–27)	22 (11–53)
Hemoglobin, g/dl	13.2 (1.6)	12.4 (1.5)
Hematocrit, ratio	0.40 (0.05)	0.38 (0.04)
ECF, %	48.2 (3.8)	48.7 (2.8)
Intracellular fluid, %	51.8 (3.8)	51.3 (2.8)
Type 2 diabetes, *n* (%)	251 (57)	51 (74)

For all continuous variables, the values between parentheses denote SD or interquartile range (B-type natriuretic peptide and *N*-terminal pro B-type natriuretic peptide). BNP, B-type natriuretic peptide; ECF, extracellular fluid; NT-proBNP, *N*-terminal pro B-type natriuretic peptide.

In a multivariable Cox regression analysis, higher zibotentan doses were significantly associated with a higher risk of fluid retention during 12 weeks of follow-up (Table 4). Participants allocated to zibotentan 0.25 mg plus dapagliflozin did not have a higher risk compared with the dapagliflozin-only arm. Participants allocated to zibotentan 5 mg plus dapagliflozin had a lower risk of fluid retention compared with zibotentan 5 mg plus placebo. In addition, the risk of fluid retention was significantly higher in patients with lower hemoglobin and higher BNP at the start of the trial. Because of the strong correlation between eGFR and hemoglobin, we also conducted a multivariable Cox regression analysis excluding hemoglobin. This model showed that a lower eGFR and a diagnosis of type 2 diabetes were independently associated with a higher risk of fluid retention (Supplemental Tables 2 and 3).

**Table 4:** Multivariable Cox proportional hazards model for fluid events

Characteristic	HR	95% CI
Age, yr	1.00	0.97 to 1.02
**Sex**		
Female	1.00 (Referent)	
Male	1.39	0.78 to 2.48
**Treatment arm**		
Dapagliflozin 10 mg	1.00 (Referent)	
Placebo	0.49	0.06 to 3.84
Zibotentan/Dapagliflozin 0.25/10 mg	1.21	0.50 to 2.91
Zibotentan/Dapagliflozin 1.5/10 mg	2.70	1.44 to 5.07
Zibotentan/Dapagliflozin 5/10 mg	3.09	1.08 to 8.80
Zibotentan 5 mg	8.50	3.40 to 21.3
Weight, kg	1.00	0.99 to 1.02
eGFR, per 5 ml/min per 1.73 m^2^	0.96	0.89 to 1.04
Hemoglobin, per g/dl	0.78	0.66 to 0.93
BNP, per doubling	1.28	1.05 to 1.55
**Type 2 diabetes**		
No	1.00 (Referent)	
Yes	1.69	0.97 to 2.97
ET-1, per doubling	1.50	0.93 to 2.43

Overall, 492 participants with complete information on covariates at baseline were included (68 fluid retention events). BNP, B-type natriuretic peptide; CI, confidence interval; ET-1, endothelin-1; HR, hazard ratio.

### Zibotentan Plasma Exposure and Fluid Retention

**Figure 3:**
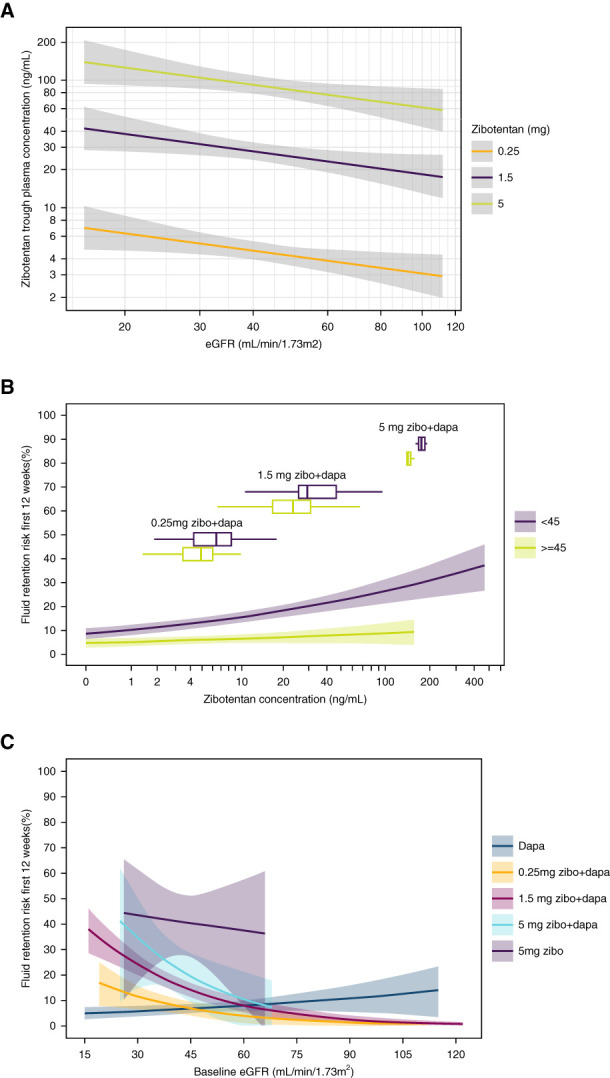
**Dose-exposure-response relationship between zibotentan and fluid retention at different levels of eGFR.** (A) Association between eGFR and zibotentan trough plasma concentration. (B) Association between baseline eGFR and the risk of fluid retention stratified by eGFR at baseline of the trial, with box plots showing the distribution of zibotentan by baseline eGFR and ZENITH-CKD treatment arm. (C) The risk of fluid retention stratified by treatment arm.

Because approximately 58% of zibotentan is excreted unchanged by the kidneys in healthy adults and both higher zibotentan doses and lower eGFR are associated with a higher risk of fluid retention, we subsequently evaluated the dose-exposure-response relationship between zibotentan and fluid retention at different levels of eGFR. As expected, zibotentan trough plasma concentration was higher at lower eGFR levels (Figure 3, A and B). Moreover, the risk of fluid retention was higher at higher zibotentan exposure (Figure 3B). This association was observed both in participants with eGFR above and below 45 ml/min per 1.73 m^2^, but the absolute risk was higher at every zibotentan exposure in participants with eGFR <45 ml/min per 1.73 m^2^. Finally, at lower eGFR with higher zibotentan doses, there was an incremental dose-dependent risk (Figure 3C).

## Discussion

In this *post hoc* analysis of data from the phase 2b ZENITH-CKD trial, we investigated the association between zibotentan treatment, alone and in combination with the SGLT2 inhibitor dapagliflozin, on fluid retention using various biomarkers and dose-response assessments. Understanding fluid dynamics during treatment with SGLT2 inhibitors and ERAs is clinically relevant as both therapies are registered for clinical use and exert complementary effects that may differentially affect fluid status. We demonstrated that early changes in body weight, BNP, and hemoglobin were independently associated with changes in bioimpedance-measured ECF in response to zibotentan treatment. Increasing doses of zibotentan as well as lower eGFR and hemoglobin and higher BNP at baseline were significantly associated with a higher risk of fluid retention during the 12-week treatment period. Impaired kidney function was associated with a higher zibotentan plasma exposure, which in turn associated with a higher risk of fluid retention. This higher risk could be attenuated by the addition of the SGLT2 inhibitor dapagliflozin.

The mechanism of fluid retention with ERA is an area of great research interest. There is some evidence that suggests that ET-1 exerts diuretic effects in the collecting duct by binding to the endothelin A (ET_A_) or B receptor (ET_B_). It has been hypothesized that ERA can cause fluid retention by directly blocking this effect through ET_A_ or indirectly blocking this effect through ET_B_ receptors at high concentrations.^7^ However, fluid retention because of antinatriuretic/antidiuretic effects would be expected to increase BP and albuminuria. In the ZENITH trial, we did not observe correlations between BP and albuminuria changes and fluid retention during the zibotentan trial, suggesting that other effects may be involved. A simulation and modeling article suggested that the antidiuresis and fluid retention during ERA treatment may instead be a response of the kidney to venodilation and increased venous capacity, which leads to a reduction in cardiac filling and cardiac output.^8^ Consequently, fluid retention occurs to restore cardiac filling and maintain cardiac output. This hypothesis could also explain why the addition of dapagliflozin to zibotentan reduced fluid retention since dapagliflozin may restore cardiac output by raising ketone bodies.^9–12^ Such effect would not be expected to occur with traditional diuretics.

Although the risk of fluid retention with high-dose zibotentan (5 mg) can be reduced when used in combination with dapagliflozin, we still observed a higher rate of fluid retention with zibotentan doses of 1.5 mg. This highlights the narrow therapeutic window and the need for careful dose selection taking into account drug and patient characteristics. Patient characteristics associated with a higher risk of fluid retention in this study, including eGFR and BNP, were consistent with prior studies. In the A Randomised, Double Blind, Placebo Controlled, Parallel Group Study to Assess the Effect of the Endothelin Receptor Antagonist Avosentan on Time to Doubling of Serum Creatinine, End Stage Renal Disease or Death in Patients With Type 2 Diabetes Mellitus and Diabetic Nephropathy study, high doses of avosentan increased the risk of heart failure, which was more frequently observed in older participants with worse glycemic control, a lower eGFR, and in those in whom body weight increased during the first weeks of treatment.^13^ In the Study Of diabetic Nephropathy with AtRasentan trial, despite careful patient selection, more fluid retention–related adverse events and heart failure hospitalizations were observed in the atrasentan compared with placebo group, in particular among women and in participants with a higher UACR, a lower eGFR, and those who experienced an early increase in BNP during atrasentan treatment.^14^

Drug characteristics should also be considered to minimize fluid retention in people with CKD. The higher zibotentan exposure at lower eGFR in the ZENITH-CKD clinical trial is in line with previous PK studies that indicated that kidney excretion is an important elimination pathway.^15–17^ The findings that both a higher zibotentan exposure and a lower eGFR are associated with a higher risk of fluid retention suggest that dose adjustments at lower eGFR is appropriate. These findings informed the study design and zibotentan dose selection of a confirmatory phase 3 randomized clinical trial, ZENITH-High Proteinuria, to evaluate the long-term efficacy of a fixed-dose combination zibotentan plus dapagliflozin compared with dapagliflozin alone in participants with CKD and high proteinuria (NCT06087835). In the ZENITH-High Proteinuria trial, the starting dose is zibotentan 0.75 mg/dapagliflozin 10 mg per day for participants with an eGFR ≥45 ml/min per 1.73 m^2^, whereas participants with an eGFR <45 ml/min per 1.73 m^2^ start with zibotentan 0.25 mg/dapagliflozin 10 mg per day. The results of the ZENITH-High Proteinuria trial will be reported when available.

We found that changes in BNP, body weight, and hemoglobin after 3 weeks of zibotentan treatment were all independently associated with changes in bioimpedance-measured ECF. An analysis of the Study Of diabetic Nephropathy with AtRasentan trial also found that both body weight and BNP were independently associated with hospitalization for heart failure during treatment with the ERA atrasentan in patients with type 2 diabetes and CKD.^14^ Independent statistically significant associations in multivariable regression suggest that these markers may reflect distinct aspects of fluid retention. BNP is released in the setting of increased ventricular wall stretch and increased atrial and ventricular pressures indicative of intravascular congestion. By contrast, peripheral edema, possibly reflected by changes in body weight, is related to presence of extravascular tissue congestion and may not directly reflect increases in cardiac filling pressure.

Because both SGLT2 inhibitors and ERAs are registered for the treatment of CKD, our study provides several relevant clinical implications on fluid dynamics with these therapies. First, patients with a lower eGFR, who may derive most benefit from ERA treatment, are at higher risk of fluid retention, highlighting the careful monitoring of biomarkers of fluid retention in this population. Second, to monitor fluid retention during ERA treatment, a combination of easily obtainable biochemical and physical markers of fluid retention, possibly reflecting different aspects of fluid retention, more accurately captures fluid retention than each marker alone. Because the risk of fluid retention was lower with combined zibotentan/dapagliflozin treatment compared with zibotentan alone, physicians should consider combination treatment of SGLT2 inhibitors with ERAs. This analysis provides a comprehensive picture of the influence of zibotentan on fluid retention in a clinical setting, using a broad range of assessments, including blood and urine-derived biomarkers, bioimpedance measurements, and PKs. The results of this study should also be interpreted in light of several limitations. The treatment arms with placebo, zibotentan 5 mg plus dapagliflozin, and zibotentan 5 mg monotherapy were halted prematurely and, therefore, concerned a limited number of participants. The trial enrolled participants with high proteinuria, and considering the multiple inclusion and exclusion criteria, the results can only be applied to people with CKD who share the characteristics of the study population. The as-treated analysis may have introduced bias by not adhering to the original randomization. Inherent to the study design, participants were only exposed to study medication for 12 weeks. Although fluid retention seems to peak after 3 weeks of treatment, longer follow-up is necessary to obtain a more comprehensive picture of the risk–benefit balance. Finally, although fluid retention was a prespecified outcome, this study was a *post hoc* analysis, and therefore, we cannot rule out chance findings.

In conclusion, we found that higher doses of zibotentan were associated with a higher risk of fluid retention, which was attenuated by combining with dapagliflozin. This risk was more pronounced at lower eGFR and higher zibotentan exposure, which highlights the importance for lower zibotentan doses with advanced stages of CKD. Careful monitoring through regular assessment of body weight, natriuretic peptides, and hemoglobin may aid in early detection of fluid retention after initiation of ERA treatment and further reduce the risk of complications. These findings may contribute to the safe and effective use of zibotentan and other ERAs in future clinical trials and clinical practice.

## Supplementary Material

## Data Availability

Partial restrictions to the data and/or materials apply. Anonymized patient-level clinical data and/or anonymized clinical study documents can be obtained through Vivli's web-based request platform (www.vivli.org). All requests will be reviewed by an independent scientific review board. AstraZeneca-sponsored studies for products that have been approved in the United States and the European Union are in scope for data requests. Additionally, AstraZeneca-sponsored studies with published primary end point results can be requested and may be considered for data and document sharing. When sharing anonymized patient-level data, complete datasets may not be shared as a result of patients withdrawing their informed consent, when clinical trial informed consent prohibits secondary use of data, or other aspects taken into consideration to protect patient privacy.

## Supplemental Material

Supplemental Table 1. Number of missing observations for fluid biomarker correlation analysis.

Supplemental Table 2. Cox proportional hazards model for fluid events, excluding hemoglobin as covariates.

Supplemental Table 3. Fluid biomarkers by baseline eGFR.

Supplemental Table 4. Change in fluid biomarkers after 3 weeks categorized by change in UACR and systolic BP.

Supplemental Figure 1. Study design.

Supplemental Figure 2. Change in selected biomarkers of fluid balance.
