# Supplementary material for: Effects of Zibotentan Alone and in Combination with Dapagliflozin on Fluid Retention in Patients with CKD
Source: J Am Soc Nephrol. 2024 Jul 12;35(10):1381–90. doi: 10.1681/ASN.0000000000000436 (PMC11452182; doi:10.1681/ASN.0000000000000436)
Supplement: Supplementary file 1 [file jasn-35-1381-s001.pdf]

ASN American Society of Nephrology | JASN Journal of the American Society of Nephrology | KIDNEY360 Accessing Our World From Every Angle | CJASN Clinical Journal of the American Society of Nephrology

ASN Journal Disclosure Form

As per ASN journal policy, I have disclosed any financial relationships or commitments I have held in the past 36 months as included below. I have listed my Current Employer below to indicate there is a relationship requiring disclosure. If no relationship exists, my Current Employer is not listed.

P. Ambery reports the following:
Employer: AstraZeneca; Ownership Interest: AstraZeneca; and Patents or Royalties: dosing patent for Cotadutide (AstraZeneca drug).

I understand that the information above will be published within the journal article, if accepted, and that failure to comply and/or to accurately and completely report the potential financial conflicts of interest could lead to the following: 1) Prior to publication, article rejection, or 2) Post-publication, sanctions ranging from, but not limited to, issuing a correction, reporting the inaccurate information to the authors' institution, banning authors from submitting work to ASN journals for varying lengths of time, and/or retraction of the published work.

Name: Philip D. Ambery
Manuscript ID: JASN-2024-000419R1
Manuscript Title: Effects of zibotentan alone and in combination with dapagliflozin on fluid retention in patients with chronic kidney disease
Date of Completion: June 8, 2024
Disclosure Updated Date: May 20, 2024

ASN American Society of Nephrology | JASN Journal of the American Society of Nephrology | KIDNEY360 Accessing Our World From Every Angle | CJASN Clinical Journal of the American Society of Nephrology

ASN Journal Disclosure Form

As per ASN journal policy, I have disclosed any financial relationships or commitments I have held in the past 36 months as included below. I have listed my Current Employer below to indicate there is a relationship requiring disclosure. If no relationship exists, my Current Employer is not listed.

M. Åstrand reports the following:
Employer: Astrazenaca R&D; Ownership Interest: Astrazenaca R&D; and Patents or Royalties: Astrazenaca R&D.

I understand that the information above will be published within the journal article, if accepted, and that failure to comply and/or to accurately and completely report the potential financial conflicts of interest could lead to the following: 1) Prior to publication, article rejection, or 2) Post-publication, sanctions ranging from, but not limited to, issuing a correction, reporting the inaccurate information to the authors' institution, banning authors from submitting work to ASN journals for varying lengths of time, and/or retraction of the published work.

Name: Magnus Åstrand
Manuscript ID: JASN-2024-000419R1
Manuscript Title: Effects of Zibotentan Alone and in Combination with Dapagliflozin on Fluid Retention in Patients with CKD
Date of Completion: June 25, 2024
Disclosure Updated Date: May 20, 2024

ASN American Society of Nephrology | JASN Journal of the American Society of Nephrology | KIDNEY360 Accessing Our World From Every Angle | CJASN Clinical Journal of the American Society of Nephrology

ASN Journal Disclosure Form

As per ASN journal policy, I have disclosed any financial relationships or commitments I have held in the past 36 months as included below. I have listed my Current Employer below to indicate there is a relationship requiring disclosure. If no relationship exists, my Current Employer is not listed.

N. Dhaun reports the following:
Employer: University of Edinburgh; Consultancy: Travere Pharmaceutical; Research Funding: I have previously received research funding from Pfizer & Travere Pharma; and Advisory or Leadership Role: Travere.

I understand that the information above will be published within the journal article, if accepted, and that failure to comply and/or to accurately and completely report the potential financial conflicts of interest could lead to the following: 1) Prior to publication, article rejection, or 2) Post-publication, sanctions ranging from, but not limited to, issuing a correction, reporting the inaccurate information to the authors' institution, banning authors from submitting work to ASN journals for varying lengths of time, and/or retraction of the published work.

Name: Neeraj Dhaun
Manuscript ID: JASN-2024-000419R1
Manuscript Title: Effects of Zibotentan Alone and in Combination with Dapagliflozin on Fluid Retention in Patients with CKD
Date of Completion: June 20, 2024
Disclosure Updated Date: June 13, 2024

ASN American Society of Nephrology · JASN Journal of the American Society of Nephrology · KIDNEY360 Accessing Our World From Every Angle · CJASN Clinical Journal of the American Society of Nephrology

ASN Journal Disclosure Form

As per ASN journal policy, I have disclosed any financial relationships or commitments I have held in the past 36 months as included below. I have listed my Current Employer below to indicate there is a relationship requiring disclosure. If no relationship exists, my Current Employer is not listed.

J. Gorriz reports the following:
Employer: Department of Nephrology. University Clinic Hospital. Valencia (Public university hospital); Consultancy: Astrazeneca, Boehringer Ingelheim, AstraZeneca, Novonordisk, Bayer, Menarini.; Research Funding: Astrazeneca, Vifor CSL; Honoraria: Honoraria for giving talks: Boehringer-Ingelheim, Astrazeneca, Menarini, Novonordisk, Novartis and Eli Lilly, Bayer.;; Advisory or Leadership Role: Astrazeneca, Boehringer-Ingelheim, Novonordisk, Bayer, Menarini; and Speakers Bureau: Boehringer-Ingelheim, Astrazeneca, Novonordisk and Eli Lilly.

I understand that the information above will be published within the journal article, if accepted, and that failure to comply and/or to accurately and completely report the potential financial conflicts of interest could lead to the following: 1) Prior to publication, article rejection, or 2) Post-publication, sanctions ranging from, but not limited to, issuing a correction, reporting the inaccurate information to the authors' institution, banning authors from submitting work to ASN journals for varying lengths of time, and/or retraction of the published work.

Name: Jose L. Gorriz
Manuscript ID: ASN-2024-000419R1
Manuscript Title: Effects of zibotentan alone and in combination with dapagliflozin on fluid retention in patients with chronic kidney disease
Date of Completion: June 8, 2024
Disclosure Updated Date: June 8, 2024

ASN American Society of Nephrology JASN Journal of the American Society of Nephrology KIDNEY360 Accessing Our World From Every Angle CJASN Clinical Journal of the American Society of Nephrology

ASN Journal Disclosure Form

As per ASN journal policy, I have disclosed any financial relationships or commitments I have held in the past 36 months as included below. I have listed my Current Employer below to indicate there is a relationship requiring disclosure. If no relationship exists, my Current Employer is not listed.

P. Greasley reports the following:
Employer: AstraZeneca; Ownership Interest: AstraZeneca; and Patents or Royalties: AstraZeneca.

I understand that the information above will be published within the journal article, if accepted, and that failure to comply and/or to accurately and completely report the potential financial conflicts of interest could lead to the following: 1) Prior to publication, article rejection, or 2) Post-publication, sanctions ranging from, but not limited to, issuing a correction, reporting the inaccurate information to the authors' institution, banning authors from submitting work to ASN journals for varying lengths of time, and/or retraction of the published work.

Name: Peter J. Greasley
Manuscript ID: JASN-2024-000419R1
Manuscript Title: Effects of Zibotentan Alone and in Combination with Dapagliflozin on Fluid Retention in Patients with CKD
Date of Completion: June 24, 2024
Disclosure Updated Date: May 21, 2024

ASN Journal Disclosure Form

As per ASN journal policy, I have disclosed any financial relationships or commitments I have held in the past 36 months as included below. I have listed my Current Employer below to indicate there is a relationship requiring disclosure. If no relationship exists, my Current Employer is not listed.

H. Heerspink reports the following:
Employer: University Medical Center Groningen; Consultancy: Ongoing consultancy agreements with AstraZeneca, Alexion, Bayer, Boehringer Ingelheim, CSL Behring, Chinook, Dimerix, Eli-Lilly, Gilead, Janssen, Novartis, NovoNordisk, Travere Pharmaceuticals; Research Funding: AstraZeneca, Boehringer Ingelheim, NovoNordisk and Janssen research support (grant funding directed to employer); Honoraria: Lecture fees from AstraZeneca and NovoNordisk; and Speakers Bureau: AstraZeneca.

I understand that the information above will be published within the journal article, if accepted, and that failure to comply and/or to accurately and completely report the potential financial conflicts of interest could lead to the following: 1) Prior to publication, article rejection, or 2) Post-publication, sanctions ranging from, but not limited to, issuing a correction, reporting the inaccurate information to the authors' institution, banning authors from submitting work to ASN journals for varying lengths of time, and/or retraction of the published work.

Name: Hiddo Jan L. Heerspink
Manuscript ID: JASN-2024-000419R1
Manuscript Title: Effects of zibotentan alone and in combination with dapagliflozin on fluid retention in patients with chronic kidney disease
Date of Completion: June 9, 2024
Disclosure Updated Date: April 22, 2024

ASN American Society of Nephrology | JASN Journal of the American Society of Nephrology | KIDNEY360 Accessing Our World From Every Angle | CJASN Clinical Journal of the American Society of Nephrology

ASN Journal Disclosure Form

As per ASN journal policy, I have disclosed any financial relationships or commitments I have held in the past 36 months as included below. I have listed my Current Employer below to indicate there is a relationship requiring disclosure. If no relationship exists, my Current Employer is not listed.

A. Mercier reports the following:
Employer: AstraZeneca; Ownership Interest: AstraZeneca; and Patents or Royalties: I am a named inventor on a patent application pending owned by AstraZeneca.

I understand that the information above will be published within the journal article, if accepted, and that failure to comply and/or to accurately and completely report the potential financial conflicts of interest could lead to the following: 1) Prior to publication, article rejection, or 2) Post-publication, sanctions ranging from, but not limited to, issuing a correction, reporting the inaccurate information to the authors' institution, banning authors from submitting work to ASN journals for varying lengths of time, and/or retraction of the published work.

Name: Anne-Kristina Mercier
Manuscript ID: JASN-2024-000419R1
Manuscript Title: Effects of zibotentan alone and in combination with dapagliflozin on fluid retention in patients with chronic kidney disease
Date of Completion: June 8, 2024
Disclosure Updated Date: May 21, 2024

ASN
American Society of Nephrology
JASN
Journal of the American Society of Nephrology
KIDNEY360
Accessing Our World From Every Angle
CJASN
Clinical Journal of the American Society of Nephrology

ASN Journal Disclosure Form

As per ASN journal policy, I have disclosed any financial relationships or commitments I have held in the past 36 months as included below. I have listed my Current Employer below to indicate there is a relationship requiring disclosure. If no relationship exists, my Current Employer is not listed.

J. Smeijer reports the following:
Employer: University Medical Center Groningen

I understand that the information above will be published within the journal article, if accepted, and that failure to comply and/or to accurately and completely report the potential financial conflicts of interest could lead to the following: 1) Prior to publication, article rejection, or 2) Post-publication, sanctions ranging from, but not limited to, issuing a correction, reporting the inaccurate information to the authors' institution, banning authors from submitting work to ASN journals for varying lengths of time, and/or retraction of the published work.

Name: Johannes David Smeijer
Manuscript ID: JASN-2024-000419R1
Manuscript Title: Effects of Zibotentan Alone and in Combination with Dapagliflozin on Fluid Retention in Patients with CKD
Date of Completion: June 21, 2024
Disclosure Updated Date: May 1, 2024

ASN American Society of Nephrology
JASN Journal of the American Society of Nephrology
KIDNEY360 Accessing Our World From Every Angle
CJASN Clinical Journal of the American Society of Nephrology

ASN Journal Disclosure Form

As per ASN journal policy, I have disclosed any financial relationships or commitments I have held in the past 36 months as included below. I have listed my Current Employer below to indicate there is a relationship requiring disclosure. If no relationship exists, my Current Employer is not listed.

M. Soler reports the following:
Employer: Hospital del Vall d´Hebron; Consultancy: Astra Zeneca; Boehringer; Esteve; Novonordisk; Mundipharma; Jansen; Travere Therapeutics, Bayer, ICU; Research Funding: Abbvie, Boehringer; Honoraria: Astra Zeneca; Boehringer; Esteve; Novonordisk; Mundipharma; Jansen; FMC, Otsuka, ICU Medical, Travere therapeutics, GSK, MEDICE; Patents or Royalties: U691ES00; Advisory or Leadership Role: CKJ; Ex-BMC Nephrology; Ex ERA-EDTA Council member, Ex SAB ERA-EDTA, Elected EIC of CKJ, Council member (vice-president) of Spanish Society of Nephrology, Kidney and Blood Pressure Research, Ex-Board ASN news, Ex-EIC of Clinical Kidney Journal, Co-Chair Western Europe ISN.; Speakers Bureau: Astra Zeneca; Boehringer; Esteve; Novonordisk; Mundipharma; Jansen; FMC, Vifor, Bayer; and Other Interests or Relationships: Sociedad Española de Nefrología. Sociedad Catalana de Nefrologia. (member); EIC of CKJ.

I understand that the information above will be published within the journal article, if accepted, and that failure to comply and/or to accurately and completely report the potential financial conflicts of interest could lead to the following: 1) Prior to publication, article rejection, or 2) Post-publication, sanctions ranging from, but not limited to, issuing a correction, reporting the inaccurate information to the authors' institution, banning authors from submitting work to ASN journals for varying lengths of time, and/or retraction of the published work.

Name: Maria Jose Soler
Manuscript ID: JASN-2024-000419R2
Manuscript Title: Effects of Zibotentan Alone and in Combination with Dapagliflozin on Fluid Retention in Patients with CKD
Date of Completion: June 25, 2024
Disclosure Updated Date: June 7, 2024

ASN American Society of Nephrology | JASN Journal of the American Society of Nephrology | KIDNEY360 Accessing Our World From Every Angle | CJASN Clinical Journal of the American Society of Nephrology

ASN Journal Disclosure Form

As per ASN journal policy, I have disclosed any financial relationships or commitments I have held in the past 36 months as included below. I have listed my Current Employer below to indicate there is a relationship requiring disclosure. If no relationship exists, my Current Employer is not listed.

V. Wasehuus reports the following:
Employer: I'm employed at Steno Diabetes Center Copenhagen; My partner has been employed at Novo Nordisk Denmark since October first 2021; and Ownership Interest: My partner has stock options in Novo Nordisk Denmark.

I understand that the information above will be published within the journal article, if accepted, and that failure to comply and/or to accurately and completely report the potential financial conflicts of interest could lead to the following: 1) Prior to publication, article rejection, or 2) Post-publication, sanctions ranging from, but not limited to, issuing a correction, reporting the inaccurate information to the authors' institution, banning authors from submitting work to ASN journals for varying lengths of time, and/or retraction of the published work.

Name: Victor Wasehuus
Manuscript ID: JASN-2024-000419R1
Manuscript Title: Effects of zibotentan alone and in combination with dapagliflozin on fluid retention in patients with chronic kidney disease
Date of Completion: June 10, 2024
Disclosure Updated Date: May 21, 2024
