# Supplementary material for: Effects of Zibotentan Alone and in Combination with Dapagliflozin on Fluid Retention in Patients with CKD
Source: J Am Soc Nephrol. 2024 Jul 12;35(10):1381–90. doi: 10.1681/ASN.0000000000000436 (PMC11452182; doi:10.1681/ASN.0000000000000436)
Supplement: Supplementary file 2 [file jasn-35-1381-s002.pdf]

Supplement to Effects of Zibotentan Alone and in Combination with Dapagliflozin on Fluid Retention in Patients with CKD

Smeijer JD, Wasehuus S, Dhaun N et.al

Table of Contents:

- Supplemental Table 1: number of missing observations for fluid biomarker correlation analysis…………………………………………………………………………………………...Page 2
- Supplemental Table 2: Cox proportional hazards model for fluid events, excluding hemoglobin as covariate………………………………………………………………………………………Page 3
- Supplemental Table 3: fluid biomarkers by baseline eGFR…………………………………Page 4
- Supplemental Table 4: change in fluid biomarkers after 3 weeks categorized by change in UACR and systolic blood pressure ……………………………………………………………Page 5
- Supplemental Figure 1: study design…………………………………………….………………Page 6
- Supplemental Figure 2: change in selected biomarkers of fluid balance …………………Page 7

Supplemental table 1A: missing variables at randomization and week 3 for the correlation of fluid biomarkers analysis among 310 participants randomized to zibotentan treatment arms

Variable	Missing, n(%)	
	Randomization	Week 3
Weight	1 (0.3)	23 (7)
Extracellular fluid	27 (9)	42 (14)
BNP	15 (5)	31 (10)
NT-proBNP	31 (10)	55 (18)
Hematocrit	37 (12)	54 (17)
Hemoglobin	36 (12)	50 (16)
Endothelin-1	25 (8)	44 (14)
UACR	28 (9)	45 (15)
Systolic blood pressure	1 (0.3)	23 (7)

Supplemental table 1B: missing variables at baseline for the multivariable Cox proportional hazards model for 508 participants who received treatment

Variable	Missing, n(%)
	Baseline
Age	0 (0.0)
Sex	0 (0.0)
Weight	0 (0.0)
eGFR	0 (0.0)
Hemoglobin	1 (0.2)
BNP	0 (0.0)
Type 2 diabetes status	0 (0.0)
Endothelin-1	15 (3)

Note: Abbreviations: BNP = B-type natriuretic peptide; NT-proBNP = N-terminal pro B-type natriuretic peptide; eGFR = estimated glomerular filtration rate; UACR = urinary albumin-creatinine ratio

Supplemental table 2: multivariable Cox proportional hazards model for fluid events, excluding hemoglobin as covariate

Characteristic	HR	95% CI
Age, years	1·00	0·97 to 1·02
Sex		
Female	1.00 (Referent)	
Male sex	1·11	0·63 to 1·95
Treatment arm		
Dapagliflozin 10mg	1.00 (Referent)	
Placebo	0·52	0·07 to 4·11
Zibotentan/Dapagliflozin 0.25/10mg	1·20	0·50 to 2·88
Zibotentan/Dapagliflozin 1.5/10mg	2·76	1·47 to 5·17
Zibotentan/Dapagliflozin 5/10mg	3·25	1·14 to 9·23
Zibotentan 5mg	8·27	3·30 to 20·7
Weight, kg	1·00	0·98 to 1·01
eGFR, per 5 mL/min/1.73m2	0·93	0·86 to 1·00
BNP, per doubling	1·31	1·08 to 1·58
Type 2 Diabetes		
No	1.00 (Referent)	
Yes	1·77	1·00 to 3·13
Endothelin-1, per doubling	1·40	0·87 to 2·25

Note: Abbreviations: BNP = B-type natriuretic peptide; eGFR = estimated glomerular filtration rate.

Supplemental table 3: ZENITH-CKD fluid biomarkers at baseline for all treatment arms by baseline: eGFR

	eGFR ≥ 45 mL/min/1.73m2		
**Characteristic**	**No, N = 298**	**Yes, N = 210**	**p-value**[1]
Weight, kg	86 (18)	85 (16)	0.235
eGFR, mL/min per 1.73m2	32 (7)	66 (19)	<0.001
BNP, ng/L	41 (21-79)	34 (18-62)	0.006
NT-proBNP, pg/mL	20 (11-42)	10 (6-21)	<0.001
Haemoglobin, g/dL	12.7 (1.5)	13.7 (1.6)	<0.001
Hematocrit, ratio	0.38 (0.05)	0.41 (0.05)	<0.001
Extracellular Fluid, %	48.6 (3.9)	47.8 (3.3)	0.009
Intracellular Fluid, %	51.4 (3.9)	52.2 (3.3)	0.009
Total Body Water, L	43.8 (9.6)	42.5 (9.2)	0.161

Note: Abbreviations: BNP = B-type natriuretic peptide; NT-proBNP = N-terminal pro B-type natriuretic peptide; eGFR = estimated glomerular filtration rate. For all continues variables the values between parentheses denote the standard deviation or interquartile range (BNP and NT-proBNP).

Supplemental table 4: ZENITH-CKD change in fluid biomarkers after 3 weeks categorized by change in UACR **(A)** and systolic blood pressure **(B)** for participants treated with zibotentan

A.

	Geom mean UACR >30% drop		
**Characteristic**	**No**, N = 100	**Yes**, N = 165	**p-value**
UACR change, log units	0.05 (0.51)	-0.88 (0.52)	<0.001
Body weight change, kg	0.18 (1.86)	0.15 (1.68)	0.901
BNP change, log units	0.13 (0.65)	-0.01 (0.66)	0.115
Hemoglobin change, g/dL	-0.75 (0.93)	-0.79 (0.72)	0.684

	Geom mean UACR >50% drop		
**Characteristic**	**No**, N = 173	**Yes**, N = 92	**p-value**
UACR change, log units	-0.19 (0.48)	-1.17 (0.54)	<0.001
Body weight change, kg	0.28 (1.84)	-0.05 (1.55)	0.126
BNP change, log units	0.10 (0.65)	-0.05 (0.66)	0.090
Hemoglobin change, g/dL	-0.73 (0.85)	-0.86 (0.72)	0.215

B.

	10 mmHg sys. blood pressure reduction		
**Characteristic**	**No, N = 187**	**Yes, N = 100**	**p-value**
Systolic blood pressure change, mmHg	1.13 (8.52)	-21.19 (8.85)	<0.001
Body weight change, kg	0.23 (1.77)	0.02 (1.59)	0.310
BNP change, log units	0.11 (0.61)	-0.18 (0.78)	0.002
Hemoglobin change, g/dL	-0.80 (0.84)	-0.70 (0.70)	0.326

	20 mmHg sys. blood pressure reduction		
**Characteristic**	**No, N = 245**	**Yes, N = 42**	**p-value**
Systolic blood pressure change, mmHg	-2.70 (10.25)	-29.67 (7.00)	<0.001
Body weight change, kg	0.14 (1.72)	0.22 (1.70)	0.790
BNP change, log units	0.05 (0.67)	-0.18 (0.76)	0.089
Hemoglobin change, g/dL	-0.75 (0.80)	-0.83 (0.74)	0.560

Note: Abbreviations: BNP = B-type natriuretic peptide; UACR = urinary albumin-creatinine ratio

Supplemental figure 1: profile of ZENITH-CKD trial. The placebo, zibotentan 5 mg and zibotentan 5 mg plus dapagliflozin 10 mg arms were discontinued. Participants randomly assigned to dapagliflozin 10 mg during part A are not shown.

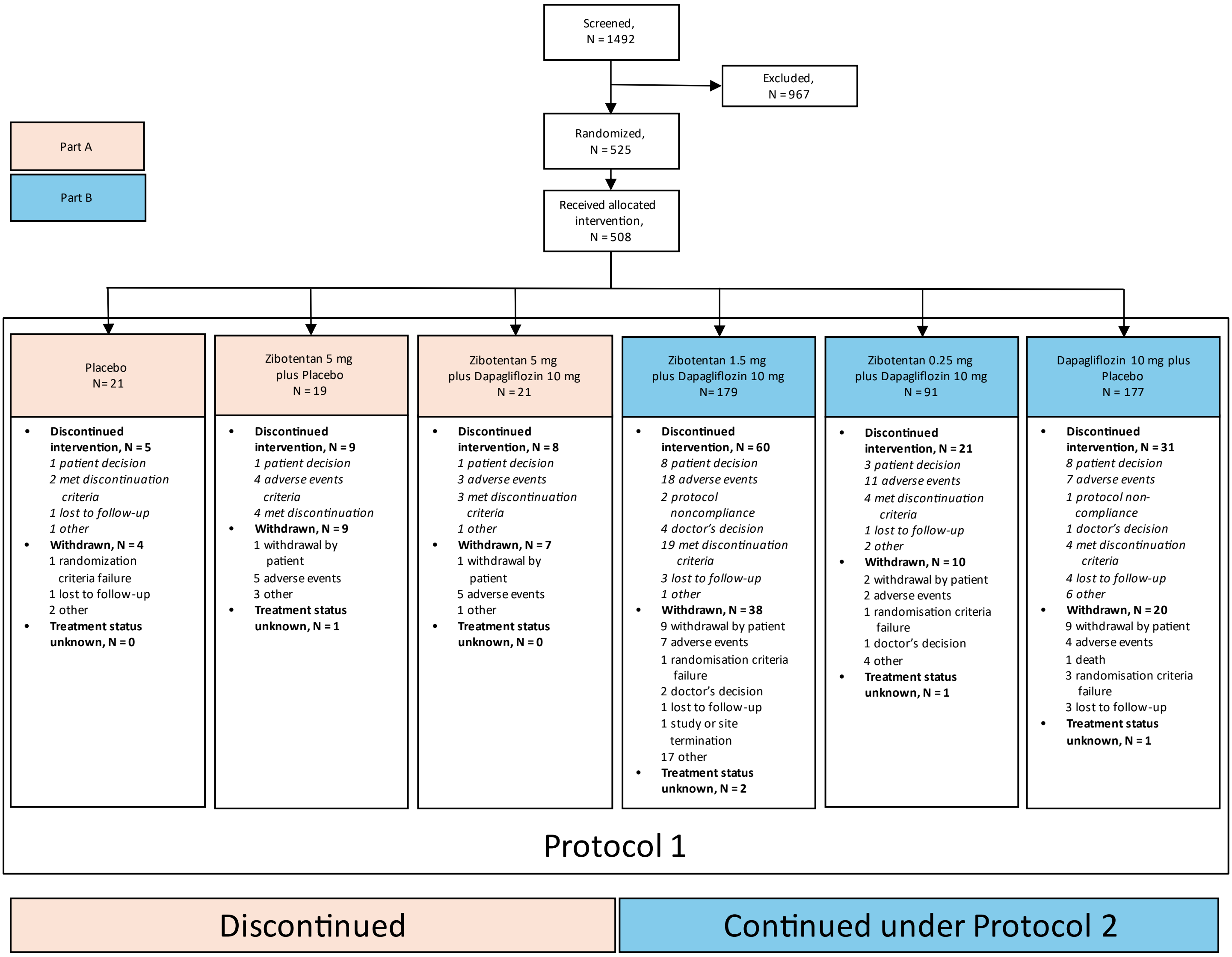

Supplemental figure 2 change in selected biomarkers of fluid balance. **A:** mean (90%CI) change in body weight from baseline; **B:** mean (90%CI) change in extracellular fluid from baseline; **C:** percentage mean (90%CI) change in BNP from baseline**; D:** mean (90%CI) change in hemoglobin from baseline;

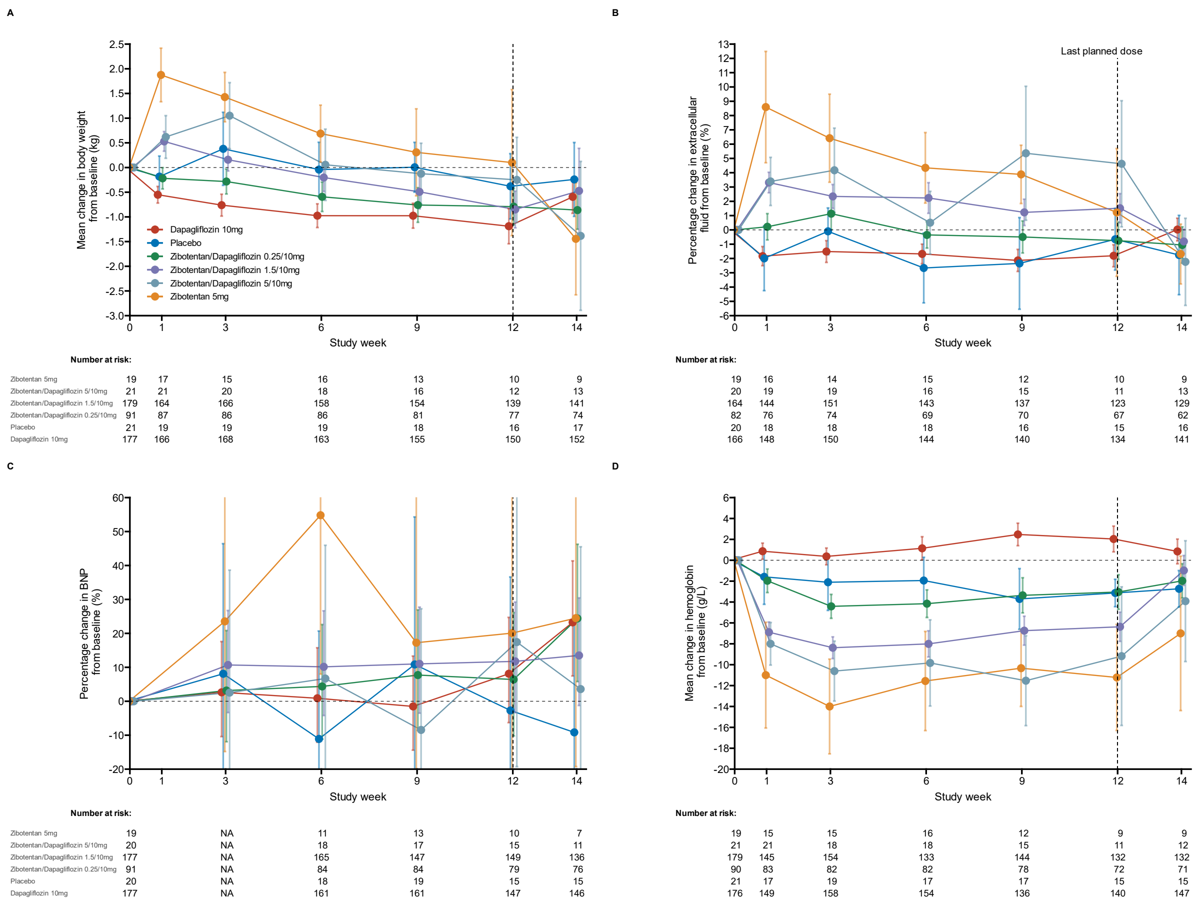

A – Number at risk:

	0	1	3	6	9	12	14
Zibotentan 5mg	19	17	15	16	13	10	9
Zibotentan/Dapagliflozin 5/10mg	21	21	20	18	16	12	13
Zibotentan/Dapagliflozin 1.5/10mg	179	164	166	158	154	139	141
Zibotentan/Dapagliflozin 0.25/10mg	91	87	86	86	81	77	74
Placebo	21	19	19	19	18	16	17
Dapagliflozin 10mg	177	166	168	163	155	150	152

B – Number at risk:

	0	1	3	6	9	12	14
	19	16	14	15	12	10	9
	20	19	19	16	15	11	13
	164	144	151	143	137	123	129
	82	76	74	69	70	67	62
	20	18	18	18	16	15	16
	166	148	150	144	140	134	141

C – Number at risk:

	0	3	6	9	12	14
Zibotentan 5mg	19	NA	11	13	10	7
Zibotentan/Dapagliflozin 5/10mg	20	NA	18	17	15	11
Zibotentan/Dapagliflozin 1.5/10mg	177	NA	165	147	149	136
Zibotentan/Dapagliflozin 0.25/10mg	91	NA	84	84	79	76
Placebo	20	NA	18	19	15	15
Dapagliflozin 10mg	177	NA	161	161	147	146

D – Number at risk:

	0	1	3	6	9	12	14
	19	15	15	16	12	9	9
	21	21	18	18	15	11	12
	179	145	154	133	144	132	132
	90	83	82	82	78	72	71
	21	17	19	17	17	15	15
	176	149	158	154	136	140	147
